# Ineficiência Ventilatória Comparável no Desempenho Máximo e Submáximo em Indivíduos com DPOC e ICC: Uma Abordagem Inovadora

**DOI:** 10.36660/abc.20230578

**Published:** 2024-04-15

**Authors:** Gerson Gatass Orro de Campos, Leandro Steinhorst Goelzer, Tiago Rodrigues de Lemos Augusto, Gisele Walter Barbosa, Gaspar R. Chiappa, Erik H. van Iterson, Paulo T. Muller

**Affiliations:** 1 Universidade Federal de Mato Grosso do Sul Hospital Maria Aparecida Pedrossian Laboratório de Fisiopatologia Respiratória Campo Grande MS Brasil Universidade Federal de Mato Grosso do Sul (UFMS) - Hospital Maria Aparecida Pedrossian (HUMAP), Laboratório de Fisiopatologia Respiratória (LAFIR), Campo Grande, MS – Brasil; 2 Universidade Evangélica de Goiás Movimento Humano e Reabilitação Anápolis GO Brasil Programa de Pós-graduação em Movimento Humano e Reabilitação, Universidade Evangélica de Goiás, Anápolis, GO – Brasil; 3 Seção de Cardiologia Preventiva e Reabilitação Clínica Cleveland MN USA Seção de Cardiologia Preventiva e Reabilitação, Clínica Cleveland, MN – EUA

**Keywords:** Doença Pulmonar Obstrutiva Crônica, Insuficiência Cardíaca, Exercício Físico, Teste de Esforço

## Abstract

**Fundamento:**

Atualmente, o excesso de ventilação tem sido fundamentado na relação entre ventilação-minuto/produção de dióxido de carbono ( ${\dot{\text{V}}}_{\text{E}}-{\dot{\text{V}}\text{CO}}_{2}$ ). Alternativamente, uma nova abordagem para eficiência ventilatória ( ${\text{η}}_{\text{E}}^{\dot{\text{V}}}$ ) tem sido publicada.

**Objetivo:**

Nossa hipótese principal é que níveis comparativamente baixos de ${\text{η}}_{\text{E}}^{\dot{\text{V}}}$ entre insuficiência cardíaca crônica (ICC) e doença pulmonar obstrutiva crônica (DPOC) são atingíveis para um nível semelhante de desempenho aeróbico máximo e submáximo, inversamente aos métodos estabelecidos há muito tempo (inclinação ${\dot{\text{V}}}_{\text{E}}-{\dot{\text{V}}\text{CO}}_{2}$ e intercepto).

**Métodos:**

Ambos os grupos realizaram testes de função pulmonar, ecocardiografia e teste de exercício cardiopulmonar. O nível de significância adotada na análise estatística foi 5%. Assim, dezenove indivíduos elegíveis para DPOC e dezenove indivíduos elegíveis para ICC completaram o estudo. Com o objetivo de contrastar valores completos de ${\dot{\text{V}}}_{\text{E}}-{\dot{\text{V}}\text{CO}}_{2}$ e ${\text{η}}_{\text{E}}^{\dot{\text{V}}}$ para o período de exercício (100%), correlações foram feitas com frações menores, como 90% e 75% dos valores máximos.

**Resultados:**

Os dois grupos tiveram características correspondentes para a idade (62±6 *vs* 59±9 anos, p>.05), sexo (10/9 *vs* 14/5, p>0,05), IMC (26±4 *vs* 27±3 Kg m^2^, p>0,05), e pico ${\dot{\text{V}}\text{O}}_{2}$ (72±19 *vs* 74±20 % pred, p>0,05), respectivamente. A inclinação ${\dot{\text{V}}}_{\text{E}}-{\dot{\text{V}}\text{CO}}_{2}$ e intercepto foram significativamente diferentes para DPOC e ICC (207,2±1,4 *vs* 33,1±5,7 e 5,3±1,9 *vs* 1,7±3,6, p<0,05 para ambas), mas os valores médios da ${\text{η}}_{\text{E}}^{\dot{\text{V}}}$ foram semelhantes entre os grupos (10,2±3,4 *vs* 10,9±2,3%, p=0,462). As correlações entre 100% do período do exercício com 90% e 75% dele foram mais fortes para ${\text{η}}_{\text{E}}^{\dot{\text{V}}}$ (r>0,850 para ambos).

**Conclusão:**

A ${\text{η}}_{\text{E}}^{\dot{\text{V}}}$ é um método valioso para comparação entre doenças cardiopulmonares, com mecanismos fisiopatológicos até agora distintos, incluindo restrições ventilatórias na DPOC.

**Figure:**
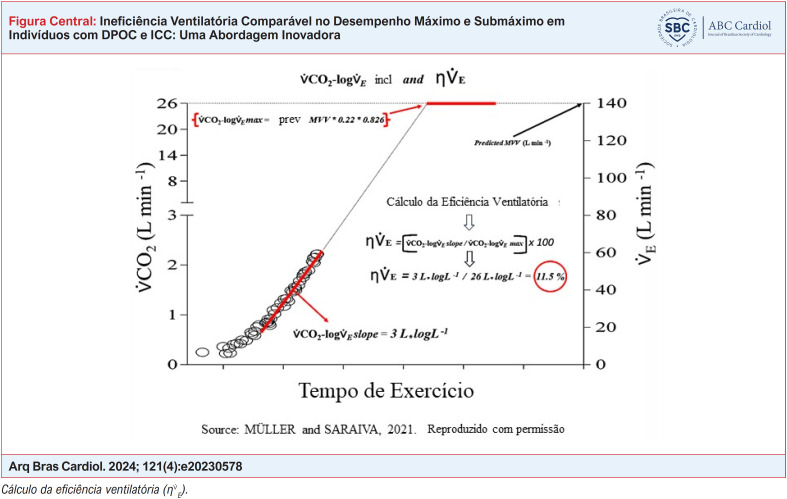


## Introdução

Quantificar o grau de eficiência ventilatória durante o teste de exercício cardiopulmonar (TECP) usando a inclinação do equivalente ventilatório para produção do dióxido de carbono ( ${\dot{\text{V}}}_{\text{E}}-{\dot{\text{V}}\text{CO}}_{2}$ ) pode ser eficaz para classificar a gravidade clínica e estimar o risco de morbidade e mortalidade dos pacientes com insuficiência cardíaca (IC).^1 - 4^ Isto ocorre porque a variável inclinação ${\dot{\text{V}}}_{\text{E}}-{\dot{\text{V}}\text{CO}}_{2}$ pode ser usada para fornecer informações se a ventilação anormalmente alta devida à demanda metabólica é provavelmente impulsionada por fatores, como a ventilação alta e perfusão discordante e/ou regulação anormal da acidose metabólica. Além disso, a inclinação do ${\dot{\text{V}}}_{\text{E}}-{\dot{\text{V}}\text{CO}}_{2}$ também reflete o excesso de ventilação secundária à capacidade oxidativa limitada e musculatura aferente hiperativada na IC, levando à hipocapnia e à exaustão precoce.^4^ No entanto, quando há doença específica afetando as vias aéreas e mecânica respiratória, como nos pacientes com doença pulmonar obstrutiva crônica (DPOC), a medição da inclinação do ${\dot{\text{V}}}_{\text{E}}-{\dot{\text{V}}\text{CO}}_{2}$ pode representar processos fisiopatológicos que provavelmente não explicarão a baixa eficiência ventilatória típica da IC.^3 - 7^

Em pacientes com DPOC, baixa eficiência ventilatória durante o TECP está comumente associada à hiperinsuflação dinâmica, alta restrição ventilatória, e restrição à expansão do volume corrente (V_T_).^5^ Este fenótipo significa que mesmo naqueles com DPOC avançada, não é raro que haja ausência de padrão de hiperventilação de IC durante o TECP,^8 - 10^ o que significa que a inclinação do ${\dot{\text{V}}}_{\text{E}}-{\dot{\text{V}}\text{CO}}_{2}$ não aumenta em conjunto com os fatores implicados com a ventilação anormalmente alta. Portanto, como fazer comparações de eficiência ventilatória entre pacientes usando a inclinação do ${\dot{\text{V}}}_{\text{E}}-{\dot{\text{V}}\text{CO}}_{2}$ pode ser desafiador, nós recentemente descrevemos um método alternativo para avaliar a eficiência ventilatória, cujo o propósito é permitir a comparação direta entre tipos de pacientes.^8^

Neste estudo, nosso objetivo foi comparar a eficiência ventilatória derivada do exercício usando nossa metodologia alternativa entre pacientes com ICC e aqueles com DPOC.^8^ Nossa hipótese é que a baixa eficiência ventilatória quantificada ao exercício usando nossa técnica alternativa é clinicamente e fisiologicamente comparáveis entre pacientes com ICC e aqueles com DPOC em ambos os níveis submáximo e máximo de consumo metabólico.^6 , 10 - 13^

## Materiais e métodos

Este estudo prospectivo e transversal foi revisado e aprovado pelo comitê de ética em pesquisa com seres humanos da Universidade Federal de Mato Grosso do Sul (UFMS) (CEP número 44517121.0.0000.0021), e atendeu aos padrões éticos e médicos de pesquisa com seres humanos descritos na Declaração de Helsinki, com provimento voluntário do consentimento informado verbal e por escrito.

### Participantes e desenho do estudo

Foram incluídos 38 participantes neste estudo que foram recrutados dos ambulatórios de clínica cardiológica e pneumológica. Os participantes foram submetidos a avaliações clínicas abrangentes e testes ao longo de três visitas do estudo, incluindo testes de função pulmonar (TFP), ecocardiografia transtorácica de repouso (ETT), e TECP no departamento de pneumologia da UFMS.

Os critérios de inclusão do estudo exigiam que os pacientes com DPOC demonstrassem uma relação volume expiratório forçado (FEV_1_)/capacidade vital forçada (CVF) menor que o Limite Inferior do Normal (LIN) e um FEV_1_<60%; ou para pacientes com ICC, os indivíduos deveriam ter demonstrado uma porcentagem da fração de ejeção do ventrículo esquerdo (FEVE) consistente com fração de ejeção reduzida ou preservada (ICFEr e ICFEp, respectivamente). Além disso, foram incluídos apenas indivíduos com presença de sinais e sintomas típicos de IC nas três categorias de história, achados físicos e raios X de tórax, após avaliação criteriosa do cardiologista, e que apresentassem estabilidade clínica. Independentemente do diagnóstico, os potenciais participantes deveriam estar estáveis clinicamente por mais de 30 dias para realizar o TECP. Os pacientes deveriam também estar livres de outras condições que poderiam ser primariamente responsáveis pelo término do TECP, como doença arterial periférica, doença restritiva pulmonar, desordens musculoesqueléticas, asma brônquica, ou bronquiectasias. Também foram excluídos os indivíduos que não conseguiram realizar os testes de estresse propostos, que eram participantes ativos de um programa de reabilitação, e que apresentavam graves intercorrências (por exemplo angina de peito). A abstinência da dependência de narcóticos e/ou álcool também foi exigida dos pacientes antes da participação neste estudo.

Indivíduos que atenderam aos critérios de inclusão no estudo realizaram TFP na primeira visita do estudo. Na segunda e terceira visita do estudo, participantes foram submetidos a ETT e TECP, respectivamente. Participantes permaneceram tomando medicamentos padrões para tratamento de DPOC ou IC nos dias de testes. No entanto, os participantes foram solicitados a se abster de tomar medicamentos depressivos/estimulantes ou ingerir cafeína nos dias de testes.

### Testes de função pulmonar

Os Participantes realizaram TFPs de acordo com as diretrizes da Sociedade Respiratória Europeia/Sociedade Torácica Americana.^14 , 15^ O mesmo sistema Vmax 22 (Viasys, Yorba Linda, USA, 2011) foi utilizado para todos os TFPs e foi calibrado antes de cada série de testes de acordo com as recomendações do fabricante e com referência a população brasileira.^16 , 17^

### Ecocardiografia doppler padrão

A ecocardiografia-doppler transtorácica de ondas pulsadas foi realizada por cardiologista com vasta experiência em adquirir imagens de pacientes com IC e DPOC. Imagens e parâmetros foram adquiridos com um equipamento padrão (Vivid S5™, General Electrics, Israel, 2015), obedecendo as diretrizes recomendadas.^18^ Os participantes ficavam na posição decúbito lateral esquerdo durante aquisição das imagens usando as incidências eixos longo paraesternal, apical quatro e duas câmaras, e subcostal. As medição das cavidades cardíacas e das espessuras do septo interventricular e parede posterior do VE foram adquiridas usando imagens no modo M. A porcentagem da fração de ejeção do VE foi quantificada usando o método Biplano de Simpson.

### Teste de exercício cardiopulmonar (TECP)

Cada TECP foi realizado num cicloergômetro modelo Vsprint 200 (Viasys, Yorba Linda, CA, USA, 2011) em um laboratório dedicado a testes clínicos de exercício . O mesmo carrinho metabólico (Vmax Encore 29, Viasys, Yorba Linda, CA, USA, 2011) foi utilizado para todos os TECP e foi calibrado antes de cada teste seguindo as orientações do fabricante.

Em pacientes com DPOC, após um período de repouso de 2 minutos e 1 minuto de fase de pedaladas sem carga a 0,0 Watts, a progressão da taxa de trabalho foi de 5 ${\text{Watts}}_{*}\min^{-1}$ para indivíduos com FEV_1_ <1,0 L or 10 ${\text{Watts}}_{*}\min^{-1}$ para aqueles com FEV_1_ ≥ 1,0 L.^19^ Em pacientes com IC, os participantes realizaram uma fase de repouso e pedaladas sem carga de modo similar aos com DPOC, que foi seguido por um exercício gradual incremental de 2 minutos numa faixa de 10 a 20 Watts.

Os dados fisiológicos foram registrados no repouso e a cada 2 minutos durante o TECP até atingir o pico do exercício, que foi definido como o tempo em que um aumento da carga de trabalho não poderia mais ser alcançada pelo ritmo de pedalada apropriada por mais de 10 segundos. Medições respiração-a-respiração do consumo de oxigênio ( ${\dot{\text{V}}\text{O}}_{2}$ ), produção de dióxido de carbono ( ${\dot{\text{V}}\text{CO}}_{\text{2}}$ ), ventilação minuto ( ${\dot{\text{V}}}_{\text{E}}$ ), frequência respiratória (fR), V_T_, etc. foram registrados durante o TECP. Frequência cardíaca (HR) e ritmo foram monitorados por meio de eletrocardiografia de 12 derivações (Cardiosoft®, USA, 2012). As medidas da saturação arterial de oxiemoglobina (SpO_2_, DIXTAL, Manaus, Brasil, 2010) foram adquiridas por meio de oximetria de pulso em repouso e durante os testes. Valores de referências ao exercício para variáveis selecionadas foram apresentadas.^20^

### Tamanho da amostra, processamento de dados e análise estatística

O tamanho da amostra foi calculado conforme previamente descrito para ICC, num estudo multicêntrico,^21^ com uma diferença média absoluta de 2.5 e um SD intra-sujeitos de 1,7 para a inclinação $\dot{\text{V}}\text{E}-{\dot{\text{V}}\text{CO}}_{2}$ . Para um desenho não pareado, isto provou que n=19 em cada grupo era um número suficiente de sujeitos para atingir um poder > 0,82% com um α=0,05. É digno de nota, para indivíduos saudáveis,^22^ um intervalo de confiança de 95% de 2,3 e um semelhante SD intra-sujeitos de 1,7 para inclinação $\dot{\text{V}}\text{E}-{\dot{\text{V}}\text{CO}}^{2}$ também provaram que n=19 em cada grupo era um número adequado de sujeitos para atingir o poder desejado (>80%).

As amostras individuais dos dados coletados foram analisadas respiração-a-respiração, sendo excluídos valores superiores a 3 vezes o desvio padrão da média local. Assim, a inclinação e o intercepto da relação ${\dot{\text{V}}}_{\text{E}}-{\dot{\text{V}}\text{CO}}_{2}$ foram obtidos por regressão linear simples do tipo: ${\dot{\text{V}}}_{\text{E}}={\text{a}}_{*}{\dot{\text{V}}\text{CO}}_{2}+/-\text{b}$ , onde "a" é a inclinação, e "b" o valor do intercepto, incluindo dados da carga do exercício até o pico.^1^ De acordo com nossa hipótese, também avaliamos dois novos parâmetros de ventilação, a taxa constante de remoção de CO_2_ ( ${\dot{\text{V}}\text{CO}}_{2}-\log{\dot{\text{V}}}_{\text{E}}$ slope) e a eficiência ventilatória ( ${\text{η}\dot{\text{V}}}_{\text{E}}$ ). As duas novas variáveis foram descritas recentemente.^8^ Resumidamente, a inclinação do ${\dot{\text{V}}\text{CO}}_{2}-\log{\dot{\text{V}}}_{\text{E}}$ foi obtida de modo semelhante àquela descrita para a inclinação da eficiência de consumo de oxigênio, ou seja, tomando o logaritmo de base 10 de ${\dot{\text{V}}}_{\text{E}}$ no eixo x contra ${\dot{\text{V}}\text{CO}}_{2}$ no eixo y. Esta relação resulta em uma função quadrática característica na maioria dos casos. O parâmetro "b" da porção linear da equação tipo ${\dot{\text{V}}\text{CO}}_{2}={\text{a}}_{*}{\dot{\text{V}}}_{\text{E}}^{2}+{\text{b}}_{*}{\dot{\text{V}}}_{\text{E}}+\text{c}$ foi denominado inclinação ${\dot{\text{V}}\text{CO}}_{2}-\log{\dot{\text{V}}}_{\text{E}}$ . Para calcular a ${\text{η}\dot{\text{V}}}_{\text{E}}$ , este valor de "b" foi tomado como uma porcentagem de um valor teórico previsto do máximo possível ${\dot{\text{V}}}_{\text{E}}$ sob condições hipotéticas, ou seja, um teto estimado de ${\dot{\text{V}}\text{CO}}_{2}$ no nível máximo previsto de ventilação voluntária (VVM) (vide Figura Central e material suplementar para mais detalhes). Esta abordagem mostrou-se mais sensível na discriminação da gravidade da obstrução e do distúrbio pulmonar difuso em indivíduos com DPOC^8^ e sem DPOC.^23^ Para efeito de comparações entre valores completos para o período de exercício (100% ou máximo), correlações foram feitas entre esses valores e frações menores, como 90% e 75% dos valores máximos.

Os dados contínuos são expressos como média±desvio padrão (DP). Variáveis categóricas foram comparadas entre os grupos por meio da estatística χ^2^ (qui-quadrado). Todas as variáveis contínuas foram analisadas quanto a distribuição pelo teste de Shapiro-Wilk. Testes t de Student não pareados foram realizados para comparação das características basais entre os grupos. O teste do coeficiente de correlação produto-momento de Pearson foi realizado para avaliar relações univariadas. A significância bicaudal foi determinada usando um nível alfa definido em 0,05. O programa estatístico SPSS 20.0 foi usado para todas as análises estatísticas (SPSS, IBM Corp®, USA, 2011).

## Resultados

### Características basais

As características demográficas e clínicas básicas de ambos os grupos de pacientes estão relatados na Tabela 1 , mostrando que os grupos foram pareados por idade, sexo, índice de massa corporal (IMC), e pico de ⩒O_2_ (%pred and mL_*_min^-1^_*_kg^-1^). A função cardíaca geral e a função pulmonar diferiram entre os grupos com IC e DPOC como esperado, enquanto os pacientes com ICC exibiram maior frequência de comorbidades. Apesar de ambos os grupos demonstrarem um nível semelhante de capacidade aeróbica, uma limitação ventilatória distinta ao exercício estava presente na DPOC. O pulso de oxigênio significativamente mais alto na IC quando comparado com DPOC foi provavelmente atribuído aos efeitos de uma maior presença de terapias limitantes da frequência deprimindo o aumento da FC na IC.

**Tabela 1:** Dados clínicos, de função pulmonar, ecocardiografia transtorácica e teste de exercício incremental (TECP) para variáveis selecionadas. Dados comparativos entre DPOC versus IC

Dados		DPOC(19)	IC (19)	Valor de p
**Características clínicas**				
	Idade(anos)	62±6	59±9	0,170
	Sexo M/F (n)	10/9	14/5	0,313
	Peso (kg)	65±15	76±12	0,003
	IMC (kg m^-^²)	26±4	27±3	0,420
	Tabagismo (p/ano)	64±41	13±21	<0,001
	mMRC/NYHA	1-4	1-3	–
	Hb(g/dlL)	15±2	14±1	0,128
**Função pulmonar**				
	FEV1 (% pred)	40±14	81±13	<0,001
	FVC (% pred)	70±17	82±14	0,040
	FEV1/FVC (%)	45±1	79±6	<0,001
	DLco (% pred)	51±21	59±18	0,650
**Ecocardiograma TT**				
	SIV Diástole (mm)	8±1	9±2	0,138
	Parede posterior (mm)	8±1	9±1	0,048
	Fração ejeção VE (%)	80±5	45±16	<0,001
	Massa VE/ASC (g/m^2^)	114±31	223±71	<0,001
**Comorbidades**				
	Hipertensão (%)	26	37	0,127
	Diabetes Mellitus (%)	0	58	<0,001
	IAM (%)	0	63	<0,001
	Aterosclerose (%)	11	37	<0,001
**Medicações**				
	SABA (%)	11	0	–
	LABA (%)	100	0	–
	LAMA (%)	26	0	–
	LAMA+LABA (%)	26	0	–
	CI (%)	42	0	<0,001
	IECA (%)	16	84	<0,001
	Betabloqueador (%)	0	95	<0,001
	Antag. aldosterone(%)	11	37	0,124
	Antidiabéticos (%)	0	58	<0,001
	Outros (%)	11	26	0,010
**TECP incremental**				
	${\dot{\text{V}}\text{O}}_{2}$ (L/min)	0,98±0,3	1,23±1,3	0,011
	${\dot{\text{V}}\text{O}}_{2}$ (%pred)	72±19	74±20	0,724
	${\dot{\text{V}}\text{O}}_{2}$ (mL/Kg/min)	15±3	16±4	0,229
	*W* (%pred)	43±17	52±20	0,110
	${\dot{\text{V}}}_{\text{E}}$ (L/min)	32±11	52±15	<0,001
	${\dot{\text{V}}}_{\text{E}}\text{/VVM}$ (%)	0,95±0,2	0,5±0,1	<0,001
	*V*_T_ (L)	1,1±0,3	1,6±0,5	0,030
	*f* R (bpm)	29±7	34±7	0,106
	FC (b/min)	126±21	120±19	0,372
	*V´* ${\dot{\text{V}}\text{O}}_{2}\text{/FC}$ (mL/batim.)	8±3	11±3	0,005

M: masculino; F: feminino; IMC: índice de massa corporal; DLCO: capacidade de difusão de monóxido de carbono; FEV1: volume expiratório forçado em 1 segundo; FVC: capacidade vital forçada; FEV1/FVC: relação forçada volume expiratório em 1 segundo e capacidade vital forçada; FC: frequência cardíaca; SIV: diâmetro do septo interventricular; IAM: infarto agudo do miocárdio; LABA: β2-agonista de longa ação antagonista betamimético de ação prolongada; LAMA: antagonista muscarínico de longa ação; VE: ventrículo esquerdo; SABA: ação curta antagonista betamimético; ${\dot{\text{V}}}_{\text{E}}$ : ventilação minuto; ${\dot{\text{V}}\text{O}}_{2}$ : captação de oxigênio; W: taxa de trabalho. Significativo p < 0,05 comparando DPOC versus IC.

### Eficiência ventilatória com desempenho máximo

**Figura 1:**
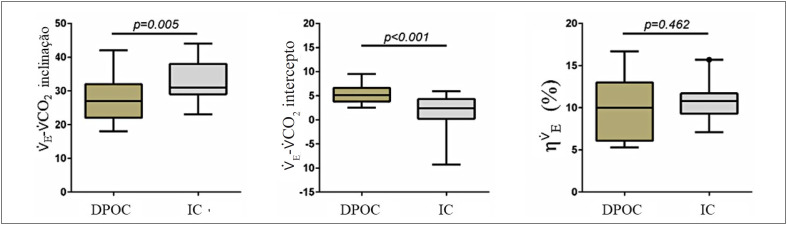
Box-plot representando a média de valores e distribuição do percentil 5-95 para inclinação $\dot{\text{V}}\text{E}-{\dot{\text{V}}\text{CO}}_{2}$ , $\dot{\text{V}}\text{E}-{\dot{\text{V}}\text{CO}}_{2}$ intercepto e ${\text{η}\dot{\text{V}}}_{\text{E}}$ , comparando DPOC vs IC.

A Tabela 2 e a Figura 1 relatam comparações de grupos para inclinação ${\dot{\text{V}}}_{\text{E}}-{\dot{\text{V}}\text{CO}}_{2}$ , ${\dot{\text{V}}}_{\text{E}}-{\dot{\text{V}}\text{CO}}_{2}$ intercepto, e ${\text{η}\dot{\text{V}}}_{\text{E}}$ . A inclinação ${\dot{\text{V}}}_{\text{E}}-{\dot{\text{V}}\text{CO}}_{2}$ e ${\dot{\text{V}}}_{\text{E}}-{\dot{\text{V}}\text{CO}}_{2}$ intercepto foram significativamente diferentes entre DPOC e ICC (27,2±1,4 vs 33,1±5,7 e 5,3±1,9 vs 1,7±3,6, p<0,05 para ambos, Figuras 1 A e 1 B , respectivamente), enquanto ${\text{η}\dot{\text{V}}}_{\text{E}}$ não diferiu significativamente entre os grupos ( Figura 1 C , p=0,462).

**Tabela 2:** Média±DP e intervalos de valores para inclinação ${\dot{\text{V}}}_{\text{E}}-{\dot{\text{V}}\text{CO}}_{2}$ , taxa constante de CO_2_ e eficiência ventilatória ( ${\text{η}\dot{\text{V}}}_{\text{E}}$ ) para indivíduos com DPOC vs IC

Variáveis	DPOC (n=19)	IC (n=19)	Valor de p
	Média±DP	Média±DP	
${\text{η}\dot{\text{V}}}_{\text{E}}$ 100, %	10,2 ± 3,4	10,9 ± 2,3	0,462
${\text{η}\dot{\text{V}}}_{\text{E}}$ 90, %	9,8 ± 3,2	10,5 ± 2,1	0,484
${\text{η}\dot{\text{V}}}_{\text{E}}$ 75, %	9,3 ± 3,0	10,3 ± 2,3	0,266
$\dot{\text{V}}{\text{CO}}_{2}-\log{\dot{\text{V}}}_{\text{E}}$ 100, $L_{*}\log L^{-1}$	2,1 ± 0,7	2,5 ± 0,6	0,100
$\dot{\text{V}}{\text{CO}}_{2}-\log{\dot{\text{V}}}_{\text{E}}$ 90, $L_{*}\log L^{-1}$	2,0 ± 0,7	2,4 ± 0,6	0,060
$\dot{\text{V}}{\text{CO}}_{2}-\log{\dot{\text{V}}}_{\text{E}}$ 75, $L_{*}\log L^{-1}$	1,9 ± 0,7	2,3 ± 0,6	0,031
${\dot{\text{V}}}_{\text{E}}{\text{/}\dot{\text{V}}\text{CO}}_{2}$ *slope*	27,2 ± 1,4	33,1 ± 5,7	0,005
${\dot{\text{V}}}_{\text{E}}{\text{/}\dot{\text{V}}\text{CO}}_{2}$ *intercept*	5,3 ± 1,9	1,7 ± 3,6	<0,001

$\dot{\text{V}}\text{CO}-\log{\dot{\text{V}}}_{\text{E}}$ : taxa constante de dióxido de carbono; ${\text{η}\dot{\text{V}}}_{\text{E}}$ : eficiência ventilatória; ${\dot{\text{V}}}_{\text{E}}$ : ventilação minuto.

### Eficiência ventilatória com desempenho submáximo

**Figura 2:**
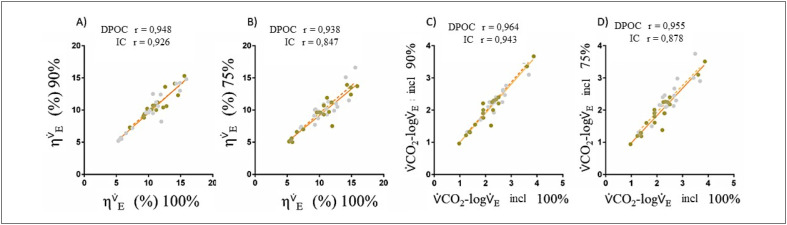
Gráficos de dispersão representando correlações entre os dados do período de 100% do exercício para ${\text{η}\dot{\text{V}}}_{\text{E}}$ e inclinação $\dot{\text{V}}\text{CO}2-\log\dot{\text{V}}\text{E}$ , e os respectivos dados submáximos de 90% e 75% do teste original completo, comparando DPOC (círculos ocres) e IC (círculos cinzas).

A Tabela 2 e Figura 2 ilustram a resposta ${\text{η}\dot{\text{V}}}_{\text{E}}$ em 100%, 90%, e 75% do tempo total do exercício, bem como a relação $\dot{\text{V}}\text{E}-{\dot{\text{V}}\text{CO}}_{2}$ . Em exercícios de intensidades submáximas, apenas a inclinação 75% ${\dot{\text{V}}\text{CO}}_{2}-\log{\dot{\text{V}}}_{\text{E}}$ foi significativamente diferente entre DPOC e ICC ( $1.9\pm 0.7{\text{L}}_{*}{\text{min}}^{-1}$ versus $2.3\pm 0.6{\text{L}}_{*}{\text{min}}^{-1}$ , respectivamente, Tabela 2 , p<0,05). No entanto, as correlações entre as medições a 100% e aquelas a 90% e 75% foram fortes para ${\text{η}\dot{\text{V}}}_{\text{E}}$ e inclinação ${\dot{\text{V}}\text{CO}}_{2}-\log{\dot{\text{V}}}_{\text{E}}$ (r>0,850 para todos, Figuras 2A, 2B, 2C e 2D , respectivamente).

### Eficiência ventilatória e pico de ${\dot{\text{V}}\text{O}}^{2}$

**Figura 3:**
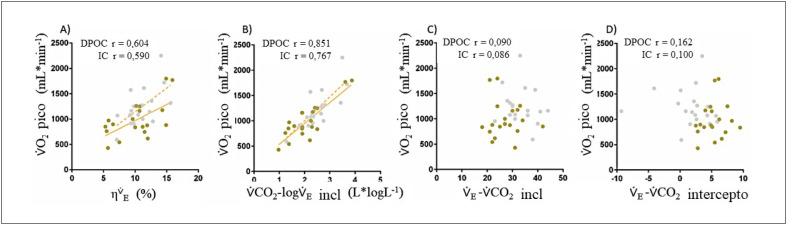
Gráficos de dispersão representando as correlações entre pico de ${\dot{\text{V}}\text{O}}_{2}$ e inclinação ${\dot{\text{V}}}_{\text{E}}-{\dot{\text{V}}\text{CO}}_{2}$ (A), ${\dot{\text{V}}}_{\text{E}}-{\dot{\text{V}}\text{CO}}_{2}$ intercepto (B), inclinação ${\dot{\text{V}}\text{CO}}_{2}-\log{\dot{\text{V}}}_{\text{E}}$ (C), e $\eta_{E}^{\dot{\text{v}}}$ (D) para DPOC (círculos ocres) e IC (círculos cinzas).

Correlações separadas envolvendo pico de ${\dot{\text{V}}\text{O}}_{2}$ e ambas ${\text{η}\dot{\text{V}}}_{\text{E}}$ e relação ${\dot{\text{V}}}_{\text{E}}-{\dot{\text{V}}\text{CO}}_{2}$ são ilustradas na Figura 3 . A força da correlação para ${\text{η}\dot{\text{V}}}_{\text{E}}$ e inclinação $\dot{\text{V}}\text{CO}2-\log\dot{\text{V}}\text{E}$ com o pico de ${\dot{\text{V}}\text{O}}_{2}$ foi moderada a alta para DPOC e IC (r=0,604/r=0,590 e r=0,851/r=0,767, p<0,001 para todos, Figuras 3 C e 3 D , respectivamente). Entretanto, correlações envolvendo a inclinação ${\dot{\text{V}}}_{\text{E}}-{\dot{\text{V}}\text{CO}}_{2}$ e ${\dot{\text{V}}}_{\text{E}}-{\dot{\text{V}}\text{CO}}_{2}$ intercepto com pico de ${\dot{\text{V}}\text{O}}_{2}$ não foram significativas (r=0,090/r=0,086, e r=0,162/r=0,100, p>0,05 para todos, Figuras 3 A e 3 B , respectivamente para DOPC/IC).

## Discussão

Este é o primeiro estudo a descrever a comparação da ${\text{η}\dot{\text{V}}}_{\text{E}}$ entre pacientes com DPOC e ICC pareados por idade, sexo e capacidade de exercício. Em contraste com as diferenças significativas entre grupos para a inclinação de ${\dot{\text{V}}}_{\text{E}}-{\dot{\text{V}}\text{CO}}_{2}$ , estes dados sugerem que ${\text{η}\dot{\text{V}}}_{\text{E}}$ não difere significativamente entre os grupos na presença de diferenças significativas para o pico de ${\dot{\text{V}}\text{O}}_{2}$ . A ${\text{η}\dot{\text{V}}}_{\text{E}}$ também demonstra moderada a forte correlação com o pico de ${\dot{\text{V}}\text{O}}_{2}$ para ambos os pacientes com DPOC e ICC, enquanto a inclinação ${\dot{\text{V}}}_{\text{E}}-{\dot{\text{V}}\text{CO}}_{2}$ não se correlaciona com o pico de ${\dot{\text{V}}\text{O}}_{2}$ para nenhum dos grupos. Assim, embora nenhuma causalidade possa ser concluída baseada no desenho do presente estudo, há utilidade clinica potencial em usar a ${\text{η}\dot{\text{V}}}_{\text{E}}$ como um marcador de eficiência ventilatória do exercício quando doença avançada que afeta as vias aéreas e mecânica respiratória provavelmente confundirá o uso de limites dos métodos tradicionais para interpretação da inclinação ${\dot{\text{V}}}_{\text{E}}-{\dot{\text{V}}\text{CO}}_{2}$ .

### Determinantes da eficiência ventilatória na IC e DPOC

Pacientes com DPOC e ICC demonstram uma infinidade de fatores fisiopatológicos que podem desencadear ventilação excessiva durante o exercício, mesmo em baixas intensidades. Dois fatores comuns que afetam a eficiência ventilatória em ambos os grupos de pacientes são um aumento na relação entre espaço morto e volume corrente (V_D_/V_T_) e um impulso neural ventilatório anormalmente alto em relação à demanda metabólica.^24^ No entanto, o efeito que esses fatores tem na diminuição da eficiência ventilatória não é tipicamente observado da mesma maneira quando comparamos a inclinação ${\dot{\text{V}}}_{\text{E}}-{\dot{\text{V}}\text{CO}}_{2}$ entre os pacientes com DPOC e ICC.

Na DPOC leve, sugere-se que a microangiopatia arterial desempenhe um papel importante no aumento da V_D_/V_T_.^25 , 26^ Entretanto, na doença avançada, sugere-se que a perda do volume do leito vascular e destruição dos espaços aéreos provocados pela exposição a longo prazo a hiperinsuflação dinâmica (HD) expandam o V_D_ total reduzindo a eficiência ventilatória.^9 , 27^ Uma diminuição do volume de reserva inspiratório também acompanha a HD, eventualmente limitando a expansão do V_T_ e contribuindo para o aumento do V_D_/V_T_. Embora o impulso neural ventilatório aumentado também possa estar presente, muitas vezes pode-se esperar que uma mecânica ventilatória perturbada silencie qualquer efeito subsequente na inclinação ${\dot{\text{V}}}_{\text{E}}-{\dot{\text{V}}\text{CO}}_{2}$ .^28 , 29^

Em contraste, em pacientes com IC, particularmente naqueles com fração de ejeção reduzida, a perda anormal da eficiência ventilatória está fortemente ligada a um estado crônico de hiperexcitação simpática decorrente de vias disfuncionais de metaborreceptores, mecanorreceptores, barorreceptores e/ou quimiorreceptores.^4 , 30 - 32^ A incapacidade adicional de V_D_/V_T_ cair e normalizar à medida que o exercício começa devido a grande e heterogênea discordância ventilação-perfusão também desempenha um papel importante na perda exagerada da eficiência ventilatória nestes pacientes.^31^

### Ineficiência ventilatória para ic e dpoc no desempenho máximo

Comparações para inclinação ${\dot{\text{V}}}_{\text{E}}-{\dot{\text{V}}\text{CO}}_{2}$ e intercepto entre DPOC e ICC tem sido reportadas na literatura de modo inconsistente, possivelmente devido a perda da concordância consistente entre a capacidade funcional e clínica (exercício) quando as comparações foram realizadas.^6 , 10^ No entanto, quando ocorreu correspondência entre os grupos, há evidências que sugerem que quando o pico de ${\dot{\text{V}}\text{O}}_{2}$ é maior que 16 ${\text{mL}}_{*}\min_{*}^{-1}{\text{kg}}^{-1}$ , a inclinação ${\dot{\text{V}}}_{\text{E}}-{\dot{\text{V}}\text{CO}}_{2}$ não difere entre DPOC e ICC.^13^ No entanto, apesar de não existir diferenças na inclinação ${\dot{\text{V}}}_{\text{E}}-{\dot{\text{V}}\text{CO}}_{2}$ entre os grupos, dado que os pacientes com DPOC demonstraram uma ${\dot{\text{V}}}_{\text{E}}-{\dot{\text{V}}\text{CO}}_{2}$ intercepto significativamente maior do que os com ICC, sugerindo que a perda da eficiência ventilatória é menos grave na DPOC do que na ICC.^13^ Por outro lado, estes dados sugerem que a eficiência ventilatória não difere entre DPOC e ICC quando comparada usando a métrica ${\text{η}\dot{\text{V}}}_{\text{E}}$ e quando os pacientes são pareados por idade, clínica, sexo e capacidade de exercício.

### Ineficiência ventilatória submáxima e pico de ${\dot{\text{V}}\text{O}}_{2}$

Estudos anteriores descreveram significativas associações entre as frações de 50%, 75% e 90% com 100% do tempo de exercício (do início ao pico) para o OUES e mostraram coeficientes de correlação semelhantes aos encontrados para ${\text{η}\dot{\text{V}}}_{\text{E}}$ .^33 , 34^ Esta pode ser mais uma forma de calcular eficiência ventilatória para fins clínicos em populações com limitações física e intelectual.^35^

A maioria dos estudos são concordantes para correlações moderadas entre ${\dot{\text{V}}}_{\text{E}}-{\dot{\text{V}}\text{CO}}_{2}$ slope e pico de ${\dot{\text{V}}\text{O}}_{2}$ para DPOC e IC,^9 , 36 - 39^ apesar de alguns resultados negativos para correlações lineares.^40 - 42^ Em indivíduos com DPOC, a predominância do fenótipo obstrutivo mais grave (GOLD III-IV) está associado com correlações fracas.^9^ A ausência de correlações significativas entre inclinação ${\dot{\text{V}}}_{\text{E}}-{\dot{\text{V}}\text{CO}}_{2}$ e pico de ${\dot{\text{V}}\text{O}}_{2}$ para DPOC e IC em nosso estudo presumivelmente resulta do intervalo estreito para ambas as variáveis em um número menor de indivíduos no estudo. No entanto, tanto a inclinação ${\dot{\text{V}}\text{CO}}_{2}-\log{\dot{\text{V}}}_{\text{E}}$ quanto a ${\text{η}\dot{\text{V}}}_{\text{E}}$ mostraram associações moderadas a fortes com o pico de ${\dot{\text{V}}\text{O}}_{2}$ . Nós especulamos que a taxa de remoção do ${\dot{\text{V}}\text{CO}}_{2}$ para cada aumento de 10 vezes no ${\dot{\text{V}}}_{\text{E}}$ está mais mecanicamente ligado à capacidade aeróbica máxima do que a relação ${\dot{\text{V}}}_{\text{E}}-{\dot{\text{V}}\text{CO}}_{2}$ , e mais estudos são necessários para elucidar os mecanismos subjacentes.

### Pontos fortes, limitações do estudo e implicações clinicas

Este estudo tem alguns pontos fortes e limitações que devem ser abordados. A nova abordagem abrangente para o cálculo da eficiência ventilatória associada com grupos bem pareados para duas doenças comuns pôde demonstrar pela primeira vez que, apesar das profundas diferenças fisiopatológicas subjacentes à relação ${\dot{\text{V}}}_{\text{E}}-{\dot{\text{V}}\text{CO}}_{2}$ anormal durante o exercício incremental, a ineficiência ventilatória pode ser muito semelhante. Isto abre um novo caminho para estudos prognósticos comparativos, por exemplo, já que a inclinação ${\dot{\text{V}}}_{\text{E}}-{\dot{\text{V}}\text{CO}}_{2}$ tem sido considerada um importante índice prognóstico para IC, mas pouco estudada em indivíduos com DPOC, dadas as limitações explicadas acima. Além disso, a possibilidade da análise submáxima da eficiência ventilatória para indivíduos com limitações físicas ou intelectuais é vantajosa. Como limitação, consideramos algumas notas desafiadoras para o cálculo do novo índice ( ${\text{η}\dot{\text{V}}}_{\text{E}}$ ). Certamente, cálculos automatizados poderiam ajudar os médicos. Neste sentido, carregamos e hospedamos códigos gratuitos do programa R para taxa constante de saída direta do CO_2_ e cálculos da ${\text{η}\dot{\text{V}}}_{\text{E}}$ (GitHub^®^).

## Conclusões

Este estudo demonstra pela primeira vez que quando a eficiência ventilatória do exercício é avaliada usando a variável ${\text{η}\dot{\text{V}}}_{\text{E}}$ e comparada entre pacientes com IC e DPOC pareados por idade, sexo e capacidade aeróbica, eficiência ventilatória não difere entre os grupos. Como a perda da eficiência ventilatória não pode ser interpretada usando os mesmos limiares de anormalidades para inclinação ${\dot{\text{V}}}_{\text{E}}-{\dot{\text{V}}\text{CO}}_{2}$ de IC para DPOC, este estudo fornece evidências preliminares que apoiam o uso da variável ${\text{η}\dot{\text{V}}}_{\text{E}}$ quando comparações de eficiência ventilatória entre grupos de pacientes levarem em conta a doença obstrutiva avançada que afeta as vias aéreas e a mecânica ventilatória. Isto poderia ser particularmente útil para a sobreposição DPOC/IC, quando teoricamente, a ineficiência ventilatória na IC poderia ser mascarada por restrições ventilatórias devido à DPOC, reduzindo o poder de avaliação prognóstica para a inclinação ${\dot{\text{V}}}_{\text{E}}-{\dot{\text{V}}\text{CO}}_{2}$ .

## *Material suplementar

Para informação adicional, por favor, clique aqui .
