# Supplementary material for: Ineficiência Ventilatória Comparável no Desempenho Máximo e Submáximo em Indivíduos com DPOC e ICC: Uma Abordagem Inovadora
Source: Arq Bras Cardiol. 2024 Apr 15;121(4):e20230578. [Article in Portuguese] doi: 10.36660/abc.20230578 (PMC11164473; doi:10.36660/abc.20230578)
Supplement: Translation

# Translation: Comparable Ventilatory Inefficiency at Maximal and Submaximal Performance in COPD vs. CHF subjects: An Innovative Approach

**DOI:** 10.36660/abc.20230578i

**Authors:** Gerson Gatass Orro de Campos, Leandro Steinhorst Goelzer, Tiago Rodrigues de Lemos Augusto, Gisele Walter Barbosa, Gaspar R. Chiappa, Erik H. van Iterson, Paulo T. Muller

**Affiliations:** 1 Campo Grande MS Brazil Universidade Federal de Mato Grosso do Sul (UFMS) - Hospital Maria Aparecida Pedrossian (HUMAP), Laboratório de Fisiopatologia Respiratória (LAFIR), Campo Grande, MS – Brazil; 2 Anápolis GO Brazil Programa de Pós-graduação em Movimento Humano e Reabilitação, Universidade Evangélica de Goiás, Anápolis, GO – Brazil; 3 Clínica Cleveland MN USA Seção de Cardiologia Preventiva e Reabilitação, Clínica Cleveland, MN – USA

**Keywords:** Chronic Obstructive Pulmonary Disease, Heart Failure, Exercise, Exercise Test

## Abstract

**Background:**

Currently, excess ventilation has been grounded under the relationship between minute-ventilation/carbon dioxide output ( ${\dot{\text{V}}}_{\text{E}}-{\dot{\text{V}}\text{CO}}_{2}$ ). Alternatively, a new approach for ventilatory efficiency ( ${\text{η}}_{\text{E}}^{\dot{\text{V}}}$ ) has been published.

**Objective:**

Our main hypothesis is that comparatively low levels of ${\text{η}}_{\text{E}}^{\dot{\text{V}}}$ between chronic heart failure (CHF) and chronic obstructive pulmonary disease (COPD) are attainable for a similar level of maximum and submaximal aerobic performance, conversely to long-established methods ( ${\dot{\text{V}}}_{\text{E}}-{\dot{\text{V}}\text{CO}}_{2}$ slope and intercept).

**Methods:**

Both groups performed lung function tests, echocardiography, and cardiopulmonary exercise testing. The significance level adopted in the statistical analysis was 5%. Thus, nineteen COPD and nineteen CHF-eligible subjects completed the study. With the aim of contrasting full values of ${\dot{\text{V}}}_{\text{E}}-{\dot{\text{V}}\text{CO}}_{2}$ and ${\text{η}\dot{\text{V}}}_{\text{E}}$ for the exercise period (100%), correlations were made with smaller fractions, such as 90% and 75% of the maximum values.

**Results:**

The two groups attained matched characteristics for age (62±6 vs. 59±9 yrs, p>.05), sex (10/9 vs. 14/5, p>0.05), BMI (26±4 vs. 27±3 Kg m^2^, p>0.05), and peak ${\dot{\text{V}}\text{O}}_{2}$ (72±19 vs. 74±20 %pred, p>0.05), respectively. The ${\dot{\text{V}}}_{\text{E}}-{\dot{\text{V}}\text{CO}}_{2}$ slope and intercept were significantly different for COPD and CHF (27.2±1.4 vs. 33.1±5.7 and 5.3±1.9 vs. 1.7±3.6, p<0.05 for both), but ${\text{η}\dot{\text{V}}}_{\text{E}}$ average values were similar between-groups (10.2±3.4 vs. 10.9±2.3%, p=0.462). The correlations between 100% of the exercise period with 90% and 75% of it were stronger for ${\text{η}\dot{\text{V}}}_{\text{E}}$ (r>0.850 for both).

**Conclusion:**

The ${\text{η}\dot{\text{V}}}_{\text{E}}$ is a valuable method for comparison between cardiopulmonary diseases, with so far distinct physiopathological mechanisms, including ventilatory constraints in COPD.

## Introduction

Quantifying the degree of ventilatory efficiency during cardiopulmonary exercise testing (CPET) using the ventilatory equivalent for carbon dioxide output ( ${\dot{\text{V}}}_{\text{E}}-{\dot{\text{V}}\text{CO}}_{2}$ ) slope can be effective for grading clinical severity and estimating morbidity and mortality risk of patients with heart failure (HF).^1 - 4^This is because the ${\dot{\text{V}}}_{\text{E}}-{\dot{\text{V}}\text{CO}}_{2}$ slope variable can be used to provide information on whether abnormally high ventilation relative to metabolic demand is likely to be driven by factors such as high ventilation and perfusion mismatch and/or abnormal regulation of metabolic acidosis. In addition, the ${\dot{\text{V}}}_{\text{E}}-{\dot{\text{V}}\text{CO}}_{2}$ slope also mirrors excess ventilation secondary to limited oxidative capacity and hyperactivated muscle afferents in HF, leading to hypocapnia and earlier exhaustion.^4^However, when a specific disease affecting the airways and breathing mechanics is present, such as in patients with chronic obstructive pulmonary disease (COPD), the ${\dot{\text{V}}}_{\text{E}}-{\dot{\text{V}}\text{CO}}_{2}$ slope measurement can represent pathophysiological processes that are unlikely to explain the low ventilatory efficiency typical of HF.^3 - 7^

In patients with COPD, low ventilatory efficiency during CPET is commonly associated with dynamic hyperinflation, high ventilatory constraint, and restricted tidal volume (V_T_) expansion.^5^This phenotype means that even in those with advanced COPD, it is not rare for there to be an absence of HF patterned hyperventilation during CPET,^8 - 10^meaning that the ${\dot{\text{V}}}_{\text{E}}-{\dot{\text{V}}\text{CO}}_{2}$ slope does not increase in tandem with the factors implicated with abnormally high ventilation. Therefore, because making cross-patient comparisons of ventilatory efficiency using the ${\dot{\text{V}}}_{\text{E}}-{\dot{\text{V}}\text{CO}}_{2}$ slope can be challenging, we recently described an alternative method for evaluating ventilatory efficiency, which is proposed to allow for direct comparison across patient types.^8^

In this study, we aimed to compare exercise ventilatory efficiency derived using our alternative methodology between patients with HF and those with COPD.^8^We hypothesized that low exercise ventilatory efficiency quantified using our alternative technique is clinically and physiologically comparable between patients with HF and those with COPD at both submaximal and maximal levels of metabolic demand.^6 , 10 - 13^

## Materials and methods

This prospective and cross-sectional study was reviewed and approved by the human research ethics committee of the Federal University of Mato Grosso do Sul (UFMS) (CEP number 44517121.0.0000.0021), and adhered to the human research medical and ethical standards outlined in the Declaration of Helsinki, with voluntary provision of verbal and written informed consent.

### Participants and study design

There were 38 participants included in this study who were recruited from outpatient cardiology and pulmonology clinics. Participants underwent comprehensive clinical evaluations and testing throughout three study visits, including pulmonary function testing (PFTs), resting transthoracic echocardiography (TTE), and CPET in the pulmonology department of the UFMS.

Study inclusion criteria required patients with COPD to demonstrate a forced expiratory volume (FEV_1_)/forced vital capacity (FVC) ratio less than the Low Limit of Normal (LLN) and an FEV_1_<60%; or for patients with HF, individuals must have demonstrated a left ventricular (LV) ejection fraction percentage consistent with either reduced or preserved ejection fraction (HFrEF and HFpEF, respectively). In addition, only subjects with the presence of signs and symptoms typical of HF in the three categories of history, physical findings, and chest X-ray after a careful cardiologist´s evaluation and who presented clinical stability were included. Regardless of diagnosis, potential participants must have been clinically stable for more than 30 days to perform CPET. Patients were also required to be free of other conditions that could have primarily accounted for the termination of CPET, such as peripheral arterial disease, restrictive pulmonary disease, musculoskeletal disorders, bronchial asthma, or bronchiectasis. Subjects who were unable to perform the proposed stress tests, who were actively participating in a rehabilitation program, and who presented severe intercurrences (e.g., *angina cordis* ) were also excluded. Abstinence of narcotic and/or alcohol dependence was also required of patients before participation in this study.

Individuals meeting study inclusion criteria performed PFTs on the first study visit. On the second and third study visits, participants underwent TTE and CPET, respectively. Participants remained on standard medications for the management of COPD or HF on testing days. However, participants were asked to abstain from taking depressant/stimulant medications or ingesting caffeine on testing days.

### Pulmonary function tests

Participants performed PFTs according to guidelines of the European Respiratory Society/American Thoracic Society.^14 , 15^The same Vmax 22 system (Viasys, Yorba Linda, USA, 2011) was used for all PFTs and was calibrated before each series of tests according to manufacturer recommendations and concerning the Brazilian population.^16 , 17^

### Standard doppler echocardiography

Transthoracic pulsed-wave Doppler-echocardiography was performed by a cardiologist-sonographer who had extensive experience in acquiring images in both HF and COPD patients. Images and parameters were acquired with a standard device (Vivid S5™, General Electrics, Israel, 2015), complying with recommended guidelines.^18^Participants were in the left lateral decubitus position during image acquisition using the parasternal long-axis, apical four and two chambers, and subcostal views. Measurement of cardiac cavities and thickness of the interventricular septum and posterior wall of the LV were acquired using M-mode imaging. The LV ejection fraction percentage was quantified using Simpson's Bi-plane method.

### Cardiopulmonary exercise testing (CPET)

Each CPET was performed on a Vsprint 200 model cycle ergometer (Viasys, Yorba Linda, CA, USA, 2011) in a dedicated clinical exercise testing laboratory. The same metabolic cart (Vmax Encore 29, Viasys, Yorba Linda, CA, USA, 2011) was used for all CPETs and was calibrated before each test according to the manufacturer's guidelines.

In patients with COPD, following a 2-min rest period and a 1-min unloaded cycling phase at 0.0 Watts, the ramp-slope work rate was 5 ${\text{Watts}}_{*}\;\min^{-1}$ for individuals with FEV_1_<1.0 L or 10 ${\text{Watts}}_{*}\;\min^{-1}$ for those with FEV_1_≥1.0 L.^19^In patients with HF, participants performed a rest and unloaded phase similar to COPD, which was followed by a 2-minute incremental step-wise exercise at a range of 10 to 20 Watts.

Physiological data were recorded at rest and every 2-min throughout CPET until achieving peak exercise, which was defined as the time point where an increase in workload could no longer be met with an appropriate pedal cadence for more than 10 seconds. Breath-by-breath measurements of oxygen uptake ( $\dot{\text{V}}{\text{O}}_{2}$ ), carbon dioxide output ( $\dot{\text{V}}{\text{CO}}_{2}$ ), minute ventilation ( ${\dot{\text{V}}}_{\text{E}}$ ), respiratory rate (fR),V_T_, etc. were recorded throughout CPET. Heart rate (HR) and rhythm were monitored via 12-lead electrocardiography (Cardiosoft^®^, USA, 2012). Measurements of arterial oxyhemoglobin saturation (SpO_2_, DIXTAL, Manaus, Brazil, 2010) were acquired using pulse oximetry at rest and throughout testing. Exercise reference values for selected variables were presented.^20^

### Sample size, data processing and statistical analysis

The sample size was calculated as previously reported for HF in a multisite study,^21^with a mean absolute difference of 2.5 and a within-subject SD of 1.7 for $\dot{\text{V}}\text{E}-\dot{\text{V}}\text{CO2}$ slope. For an unpaired design, this proved that n=19 in each group was a sufficient number of subjects to reach a power > 0.82% with an α=0.05. Of note, for healthy subjects,^22^a 95% confidence interval of 2.3 and a similar within-subject SD of 1.7 for $\dot{\text{V}}\text{E}-\dot{\text{V}}\text{CO2}$ slope also proved that n=19 in each group was an adequate number of subjects to reach the desired power (>80%).

The individual collected data samples were analyzed breath-by-breath, with values exceeding 3 times the standard deviation of the local average being excluded. Thus, the slope and intercept of the ${\dot{\text{V}}}_{\text{E}}-\dot{\text{V}}{\text{CO}}_{2}$ ratio were obtained by simple linear regression of the type: ${\dot{\text{V}}}_{\text{E}}={\text{a}}_{*}{\text{VCO}}_{2}+/-\text{b}$ , where "a" is the slope, and "b" is the value of the intercept, including data from loading exercise to peak.^1^In agreement with our hypothesis, we also evaluated two new ventilation parameters: the CO_2_output constant rate ( $\dot{\text{V}}{\text{CO}}_{2}-\log{\dot{\text{V}}}_{\text{E}}$ slope) and the ventilatory efficiency ( ${\text{η}\dot{\text{V}}}_{\text{E}}$ ). The two new variables have been described recently.^8^Briefly, the $\dot{\text{V}}{\text{CO}}_{2}-\log{\dot{\text{V}}}_{\text{E}}$ slope was obtained in a manner similar to that described for the oxygen uptake efficiency slope, that is, taking the base 10^th^logarithm of ${\dot{\text{V}}}_{\text{E}}$ on the x-axis against $\dot{\text{V}}{\text{CO}}_{2}$ on the y-axis. This relationship results in a characteristic quadratic function in most cases. The parameter "b" of the linear part of the equation type ${\dot{\text{V}}\text{CO}}_{2}={\text{a}}_{*}{\dot{\text{V}}}_{\text{E}}^{2}+{\text{b}}_{*}{\dot{\text{V}}}_{\text{E}}+\text{c}$ was termed ( $\dot{\text{V}}{\text{CO}}_{2}-\log{\dot{\text{V}}}_{\text{E}}$ slope). To calculate ${\text{η}\dot{\text{V}}}_{\text{E}}$ , this value of "b" was taken as a percentage of a predicted theoretical value of maximum possible ${\dot{\text{V}}}_{\text{E}}$ under hypothetical conditions, that is, an estimated ceiling of $\dot{\text{V}}{\text{CO}}_{2}$ at the predicted maximal voluntary ventilation (MVV) level (see Central Figure and supplementary material for more details). This approach proved to be more sensitive to the discrimination of severity of obstruction and diffusive pulmonary disorder in individuals with COPD^8^and smokers without COPD.^23^For comparisons between full values for the exercise period (100% or maximum), correlations were made between these values and smaller fractions, such as 90% and 75% of the maximum values.

Continuous data are expressed as mean±standard deviation (SD). Categorical variables were compared between groups using the χ^2^(chi-square) statistic. All continuous variables were analyzed for distribution by the Shapiro-Wilk test. Unpaired Student's t-tests were performed for the comparison of baseline characteristics between groups. Pearson product-moment correlation coefficient testing was performed to evaluate univariate relationships. Two-tailed significance was determined using an alpha level set at 0.05. The statistical program SPSS 20.0 was used for all statistical analyses (SPSS, IBM Corp®, USA, 2011).

## Results

### Baseline characteristics

Basic demographic and clinical characteristics for both patient groups are reported in Table 1 , showing groups were matched for age, sex, body mass index (BMI), and peak ⩒O_2_ (%pred and mL_*_min^-1^_*_kg^-1^). Overall cardiac function and pulmonary function differed between HF and COPD groups as expected, whereas HF patients exhibited a higher frequency of comorbidities. Despite both groups demonstrating a similar level of aerobic capacity, a distinct ventilatory limitation to exercise was present in COPD. The significantly higher oxygen pulse in HF as compared with COPD was likely attributable to effects from a greater presence of rate-limiting therapies depressing the rise in HR in HF.

**Table 1:** Clinical, Lung function, TT echocardiographic, and Incremental Exercise Testing (CPET) data for selected variables. Comparative data between COPD versus HF

Data		COPD(19)	HF (19)	p-value
**Clinical features**				
	Age(yrs)	62±6	59±9	0.170
	Gender M/F (n)	10/9	14/5	0.313
	Weight (kg)	65±15	76±12	0.003
	BMI (kg m^-^²)	26±4	27±3	0.420
	Smoking (p/y)	64±41	13±21	<0.001
	mMRC/NYHA	1-4	1-3	–
	Hb(g/dlL)	15±2	14±1	0.128
**Lung function**				
	FEV1 (% pred)	40±14	81±13	<0.001
	FVC (% pred)	70±17	82±14	0.040
	FEV1/FVC (%)	45±1	79±6	<0.001
	DLco (% pred)	51±21	59±18	0.650
**TT Echocardiography**				
	Diastolic IVS (mm)	8±1	9±2	0.138
	Posterior wall (mm)	8±1	9±1	0.048
	LV Ejection fraction (%)	80±5	45±16	<0.001
	LV mass/BSA (g/m^2^)	114±31	223±71	<0.001
**Comorbidity**				
	SA Hypertension (%)	26	37	0.127
	Diabetes Mellitus (%)	0	58	<0.001
	AMI (%)	0	63	<0.001
	Atherosclerosis (%)	11	37	<0.001
**Medications**				
	SABA (%)	11	0	–
	LABA (%)	100	0	–
	LAMA (%)	26	0	–
	LAMA+LABA (%)	26	0	–
	IC (%)	42	0	<0.001
	ACE (%)	16	84	<0.001
	Betablock (%)	0	95	<0.001
	Aldosterone antagonist (%)	11	37	0.124
	Antidiabetic drug (%)	0	58	<0.001
	Other (%)	11	26	0.010
**Incremental CPET**				
	$\dot{\text{V}}{\text{O}}_{2}$ (L/min)	0.98±0.3	1.23±1.3	0.011
	$\dot{\text{V}}{\text{O}}_{2}$ (%pred)	72±19	74±20	0.724
	$\dot{\text{V}}{\text{O}}_{2}$ (mL/Kg/min)	15±3	16±4	0.229
	W (%pred)	43±17	52±20	0.110
	${\dot{\text{V}}}_{\text{E}}$ (L/min)	32±11	52±15	<0.001
	${\dot{\text{V}}}_{\text{E}}\text{/MVV}$ (%)	0.95±0.2	0.5±0.1	<0.001
	V_T_(L)	1.1±0.3	1.6±0.5	0.030
	fR (bpm)	29±7	34±7	0.106
	HR (beats/min)	126±21	120±19	0.372
	V´O_2_/HR (mL/beat)	8±3	11±3	0.005

BMI: body mass index; DL_CO_: diffusing capacity for carbon monoxide; FEV_1:_forced expiratory volume in 1 s; FVC: forced vital capacity; FEV_1_/FVC: ratio on forced expiratory volume in 1 s and forced vital capacity; HR: heart-rate; IVS: interventricular septum diameter; AMI: acute myocardial infarct; LABA: long-acting β_2_-agonist long-action betamimetic antagonist; LAMA: long-acting muscarinic antagonist; LV: left ventricular; mMRC, modified Medical Research Council; SABA: short-action betamimetic antagonist; ${\dot{\text{V}}}_{\text{E}}$ : minute-ventilation; $\dot{\text{V}}{\text{O}}_{2}$ : oxygen uptake; W: work-rate. Significant *p* < 0.05 comparing COPD *vs.* HF.

### Ventilatory efficiency at maximal performance

Table 2 and Figure 1 report group comparisons for ${\dot{\text{V}}}_{\text{E}}-{\dot{\text{V}}\text{CO}}_{2}$ slope, ${\dot{\text{V}}}_{\text{E}}-{\dot{\text{V}}\text{CO}}_{2}$ intercept, and ${\text{η}\dot{\text{V}}}_{\text{E}}$ . The ${\dot{\text{V}}}_{\text{E}}-{\dot{\text{V}}\text{CO}}_{2}$ slope and ${\dot{\text{V}}}_{\text{E}}-{\dot{\text{V}}\text{CO}}_{2}$ intercept were significantly different between COPD and HF (27.2±1.4 vs. 33.1±5.7 and 5.3±1.9 vs. 1.7±3.6, p<0.05 for both, Figures 1 A and 1 B , respectively), whereas ${\text{η}\dot{\text{V}}}_{\text{E}}$ did not differ significantly between groups ( Figure 1 C , p=0.462).

**Table 2:** Average±SD and range values for $\dot{\text{V}}\text{E}-\dot{\text{V}}\text{CO2}$ slope, CO2 constant-rate, and ventilatory efficiency ( $\text{η}\dot{\text{V}}\text{E}$ ) for COPD vs. HF subjects

Variable	COPD (n=19)	HF (n=19)	p-value
	Average±SD	Average±SD	
${\text{η}\dot{\text{V}}}_{\text{E}}$ 100, %	10.2 ± 3.4	10.9 ± 2.3	0.462
${\text{η}\dot{\text{V}}}_{\text{E}}$ 90, %	9.8 ± 3.2	10.5 ± 2.1	0.484
${\text{η}\dot{\text{V}}}_{\text{E}}$ 75, %	9.3 ± 3.0	10.3 ± 2.3	0.266
$\dot{\text{V}}{\text{CO}}_{2}-\log{\dot{\text{V}}}_{\text{E}}$ 100, $L_{*}\log L^{-1}$	2.1 ± 0.7	2.5 ± 0.6	0.100
$\dot{\text{V}}{\text{CO}}_{2}-\log{\dot{\text{V}}}_{\text{E}}$ 90, $L_{*}\log L^{-1}$	2.0 ± 0.7	2.4 ± 0.6	0.060
$\dot{\text{V}}{\text{CO}}_{2}-\log{\dot{\text{V}}}_{\text{E}}$ 75, $L_{*}\log L^{-1}$	1.9 ± 0.7	2.3 ± 0.6	0.031
${\dot{\text{V}}}_{\text{E}}\text{/}\dot{\text{V}}{\text{CO}}_{2}$ *slope*	27.2 ± 1.4	33.1 ± 5.7	0.005
${\dot{\text{V}}}_{\text{E}}\text{/}\dot{\text{V}}{\text{CO}}_{2}$ *intercept*	5.3 ± 1.9	1.7 ± 3.6	<0.001

$\dot{\text{V}}\text{CO2}-\log\dot{\text{V}}\text{E}$ : carbon dioxide constant-rate; $\text{η}\dot{\text{V}}\text{E}$ : ventilatory efficiency; $\dot{\text{V}}\text{E}$ : Minute-ventilation.

**Figure 1:** Box-plot depicting average values and 5-95 percentile distribution for ${\dot{\text{V}}}_{\text{E}}-{\dot{\text{V}}\text{CO}}_{2}$ slope, ${\dot{\text{V}}}_{\text{E}}-{\dot{\text{V}}\text{CO}}_{2}$ intercept, and ${\text{η}\dot{\text{V}}}_{\text{E}}$ in comparing COPD vs. HF.

### Ventilatory efficiency at submaximal performance

Table 2 and Figure 2 illustrate the ${\text{η}\dot{\text{V}}}_{\text{E}}$ response at 100%, 90%, and 75% of the total exercise time frame, as well as the ${\dot{\text{V}}}_{\text{E}}-{\dot{\text{V}}\text{CO}}_{2}$ relationship. At submaximal exercise intensities, only 75% $\dot{\text{V}}{\text{CO}}_{2}-\log{\dot{\text{V}}}_{\text{E}}$ slope was significantly different between COPD and HF ( $1.9\pm 0.7{\text{L}}_{*}{\text{min}}^{-1}$ versus $2.3\pm 0.6{\text{L}}_{*}\min^{-1}$ , respectively, Table 2 , p<0.05). However, correlations between measurements at 100% and those at 90% and 75% were strong for ${\text{η}\dot{\text{V}}}_{\text{E}}$ and $\dot{\text{V}}{\text{CO}}_{2}-\log{\dot{\text{V}}}_{\text{E}}$ slope (r>0.850 for all, Figures 2 A, 2B, 2C and 2D , respectively).

**Figure 2:** Scatter-plots representing correlations between 100% exercise time-frame data for ${\text{η}}^{\dot{\text{V}}}\text{E}$ and $\dot{\text{V}}{\text{CO}}_{2}-\log{\dot{\text{V}}}_{\text{E}}$ slope, and the respective 90% and 75% submaximal data of the original complete test, in comparing COPD (Ochre circles) and HF (Grey circles).

### Ventilatory efficiency and $\dot{\text{V}}{\text{O}}_{2}$ peak

Separate correlations involving $\dot{\text{V}}{\text{O}}_{2}$ peak and both ${\text{η}\dot{\text{V}}}_{\text{E}}$ and ${\dot{\text{V}}}_{\text{E}}-{\dot{\text{V}}\text{CO}}_{2}$ relationship are illustrated in Figure 3 . Correlation strength for ${\text{η}\dot{\text{V}}}_{\text{E}}$ and $\dot{\text{V}}{\text{CO}}_{2}-\log{\dot{\text{V}}}_{\text{E}}$ slope with $\dot{\text{V}}{\text{O}}_{2}$ peak was moderate-to-high for COPD and HF (r=0.604/r=0.590 and r=0.851/r=0.767, p<0.001 for all, Figures 3 C and 3 D , respectively). However, correlations involving the ${\dot{\text{V}}}_{\text{E}}-{\dot{\text{V}}\text{CO}}_{2}$ slope and ${\dot{\text{V}}}_{\text{E}}-{\dot{\text{V}}\text{CO}}_{2}$ intercept with $\dot{\text{V}}{\text{O}}_{2}$ peak were not significant (r=0.090/r=0.086, and r=0.162/r=0.100, p>0.05 for all, Figures 3 A and 3 B , respectively for COPD/HF).

**Figure 3:** Scatter-plots representing correlations between $\dot{\text{V}}\text{O2}$ peak and $\dot{\text{V}}\text{E}-\dot{\text{V}}\text{CO2}$ slope (A), $\dot{\text{V}}\text{E}-\dot{\text{V}}\text{CO2}$ intercept (B), $\dot{\text{V}}\text{CO2}-\log\dot{\text{V}}\text{E}$ slope (C), and $\text{η}\dot{\text{V}}\text{E}$ (D) for COPD (Ochre circles) and HF (Grey circles).

## Discussion

This is the first study to describe the comparison of ${\text{η}\dot{\text{V}}}_{\text{E}}$ between COPD and HF patients matched for age, sex, and exercise capacity. In contrast to the significant differences between groups for the ${\dot{\text{V}}}_{\text{E}}-{\dot{\text{V}}\text{CO}}_{2}$ slope, these data suggest that ${\text{η}\dot{\text{V}}}_{\text{E}}$ does not significantly differ between groups in the presence of no significant differences for $\dot{\text{V}}{\text{O}}_{2}$ peak. The ${\text{η}\dot{\text{V}}}_{\text{E}}$ also demonstrates a moderate-to-strong correlation with $\dot{\text{V}}{\text{O}}_{2}$ peak for both COPD and HF patients, whereas the ${\dot{\text{V}}}_{\text{E}}-{\dot{\text{V}}\text{CO}}_{2}$ slope does not correlate with $\dot{\text{V}}{\text{O}}_{2}$ peak for either group. Thus, although no causality can be concluded based on the present study design, there is potential clinical utility in using the ${\text{η}\dot{\text{V}}}_{\text{E}}$ as a marker of exercise ventilatory efficiency when advanced disease affecting the airways and ventilatory mechanics is likely to confound the use of traditional thresholds for interpreting the ${\dot{\text{V}}}_{\text{E}}-{\dot{\text{V}}\text{CO}}_{2}$ slope.

### Determinants of ventilatory efficiency in HF and COPD

Patients with COPD or HF demonstrate a multitude of pathophysiological factors that can trigger excessive ventilation during exercise, even at low intensities. Two common factors affecting ventilatory efficiency in both patient groups are an increase in the dead space to tidal volume ratio (V_D_/V_T_) and abnormally high ventilatory neural drive relative to metabolic demand.^24^However, the effect these factors have on lessening ventilatory efficiency is not typically observed in the same manner when comparing the ${\dot{\text{V}}}_{\text{E}}-{\dot{\text{V}}\text{CO}}_{2}$ slope between COPD and HF patients.

In mild COPD, arterial microangiopathy is suggested to play a major role in the increase in V_D_/V_T._^25 , 26^However, in advanced disease, it is suggested that the loss of vascular bed volume and destruction of air spaces provoked by long-term exposure to dynamic hyperinflation (DH) expands total V_D_to lessen ventilatory efficiency.^9 , 27^A decrease in inspiratory reserve volume also follows DH, eventually limiting V_T_expansion and contributing to the increase in V_D_/V_T_. Although increased ventilatory neural drive can also be present, deranged ventilatory mechanics can often be expected to mute any subsequent effect on the ${\dot{\text{V}}}_{\text{E}}-{\dot{\text{V}}\text{CO}}_{2}$ slope.^28 , 29^

In contrast, in patients with HF, particularly in those with reduced ejection fraction, the abnormal loss of ventilatory efficiency is strongly linked to a chronic state of hyper-sympathoexcitation stemming from dysfunctional metaboreceptor, mechanoreceptor, baroreceptor, and/or chemoreceptor pathways.^4 , 30 - 32^The additional inability of V_D_/V_T_to fall and normalize as exercise commences because of high and heterogeneous ventilation-to-perfusion mismatching also plays an important role in the exaggerated loss of ventilatory efficiency in these patients.^31^

### Ventilatory inefficiency for hf and copd at maximal performance

Comparisons for the ${\dot{\text{V}}}_{\text{E}}-{\dot{\text{V}}\text{CO}}_{2}$ slope and intercept between COPD and HF have been inconsistently reported in the literature, possibly because there has been a lack of consistent clinical and functional capacity (exercise) matching when comparisons have been performed.^6 , 10^However, when group matching has occurred, there is evidence to suggest that when ⩒O_2_ peak is greater than 16 mL_*_min^-1^_*_kg^-1^, the ${\dot{\text{V}}}_{\text{E}}-{\dot{\text{V}}\text{CO}}_{2}$ slope does not differ between COPD and HF.^13^However, despite there being no differences in the ${\dot{\text{V}}}_{\text{E}}-{\dot{\text{V}}\text{CO}}_{2}$ slope between groups, given that COPD patients demonstrated a significantly higher ${\dot{\text{V}}}_{\text{E}}-{\dot{\text{V}}\text{CO}}_{2}$ intercept than HF, it is suggested that the loss of ventilatory efficiency is less severe in COPD than in HF.^13^By contrast, these data suggest that ventilatory efficiency does not differ between COPD and HF when compared using the ${\text{η}\dot{\text{V}}}_{\text{E}}$ metric and when patients are matched for clinical age, sex, and exercise capacity.

### Submaximal ventilatory inefficiency and $\dot{\text{V}}{\text{O}}_{2}$ peak

Previous studies described significant associations between fractions of 50%, 75%, and 90% with 100% exercise time (from start to peak) for the OUES and showed coefficients of correlation similar to that found for ${\text{η}\dot{\text{V}}}_{\text{E}}$ ^33 , 34^This may be one more way of calculating ventilatory efficiency in physically or intellectually limited populations for clinical purposes.^35^

The majority of studies are concordant for moderate correlations between ${\dot{\text{V}}}_{\text{E}}-{\dot{\text{V}}\text{CO}}_{2}$ slope and $\dot{\text{V}}{\text{O}}_{2}$ peak for COPD and HF,^9 , 36 - 39^despite some negative results for linear correlations.^40 - 42^In COPD subjects, the predominance of more severe obstructive phenotype (GOLD III-IV) is associated with weaker correlations.^9^The absence of significant correlations between ${\dot{\text{V}}}_{\text{E}}-{\dot{\text{V}}\text{CO}}_{2}$ slope and $\dot{\text{V}}{\text{O}}_{2}$ peak for COPD and HF in our study presumably results from the narrow range for both variables in a smaller number of subjects in the study. However, both $\dot{\text{V}}{\text{CO}}_{2}-\log{\dot{\text{V}}}_{\text{E}}$ slope and ${\text{η}\dot{\text{V}}}_{\text{E}}$ showed moderate-to-strong associations with $\dot{\text{V}}{\text{O}}_{2}$ peak. We speculate that the rate of $\dot{\text{V}}{\text{CO}}_{2}$ clearance for each 10-fold increase in ${\dot{\text{V}}}_{\text{E}}$ is more mechanistically linked to maximal aerobic capacity than ${\dot{\text{V}}}_{\text{E}}-{\dot{\text{V}}\text{CO}}_{2}$ relationship, and further studies are warranted to elucidate the underpinning mechanisms.

### Strengths, limitations of the study and clinical implications

This study has some strengths and limitations that should be addressed. The new comprehensive approach for ventilatory efficiency calculation associated with well-paired groups for two common diseases could be shown for the first time that, despite profound pathophysiological differences underpinning abnormal ${\dot{\text{V}}}_{\text{E}}-{\dot{\text{V}}\text{CO}}_{2}$ relationship during the incremental exercise, ventilatory inefficiency might be very similar. This opens a new avenue for comparative prognostic studies, for instance, as ⩒ the _E_-⩒CO_2_ slope has been considered an important prognostic index for HF but scarcely studied for COPD subjects given the above-explained limitations. Moreover, the possibility of submaximal analysis of the ventilatory efficiency for physically or intellectually limited subjects is advantageous. As a limitation, we consider some grades challenging for the calculation of the new index ( ${\text{η}\dot{\text{V}}}_{\text{E}}$ ). Certainly, automatized calculations could help clinicians. In this sense, we have uploaded and hosted free R-program codes for direct CO_2_output constant rate and ${\text{η}\dot{\text{V}}}_{\text{E}}$ calculations (GitHub^®^).

## Conclusions

This study demonstrates for the first time that when exercise ventilatory efficiency is evaluated using the ${\text{η}\dot{\text{V}}}_{\text{E}}$ variable and compared between patients with HF and COPD matched for age, sex, and aerobic capacity, ventilatory efficiency does not differ between groups. Because the loss of ventilatory efficiency cannot be interpreted using the same thresholds of abnormality for the ${\dot{\text{V}}}_{\text{E}}-{\dot{\text{V}}\text{CO}}_{2}$ slope from HF to COPD, this study provides preliminary evidence supporting the use of the ${\text{η}\dot{\text{V}}}_{\text{E}}$ variable when comparisons of ventilatory efficiency between patient groups must account for advanced obstructive disease affecting the airways and ventilatory mechanics. This could be particularly useful for COPD/HF overlapping when, theoretically, the ventilatory inefficiency in HF could be masked by ventilatory constraints due to COPD, reducing the power of the prognostic evaluation for ${\dot{\text{V}}}_{\text{E}}-{\dot{\text{V}}\text{CO}}_{2}$ slope.

## *Supplemental Materials

For additional information, please click here .
